# The Antisense long noncoding RNA AGAP2-AS1 regulates cell proliferation and metastasis in Epithelial Ovarian Cancer

**DOI:** 10.7150/jca.36636

**Published:** 2020-07-09

**Authors:** Zheng Tingting, Lin Xiaojing, Tang Xiaoyan, Hua Keqin, Qiu Junjun

**Affiliations:** 1 Department of Gynaecology, Obstetrics and Gynaecology Hospital, Fudan University, 419 Fangxie Road, Shanghai 200011, China Shanghai 200011, China.; 2 Department of Obstetrics and Gynaecology of Shanghai Medical College, Fudan University, 138 Yixueyuan Road, Shanghai 200032, China.; 3 Shanghai Key Laboratory of Female Reproductive Endocrine-Related Diseases, 413 Zhaozhou Road, Shanghai 200011, China.; 4 Department of Gynaecology, The First affiliated Hospital of Zhengzhou University, Zhengzhou University, 1 Jianshe Road, Zhengzhou 471000, Zhengzhou 471000, China.

**Keywords:** AGAP2-AS1, proliferation, metastasis, epithelial ovarian cancer

## Abstract

Antisense long noncoding RNAs serve as important regulators of protein-coding genes and contribute to tumorigenesis and metastasis. AGAP2-AS1, an antisense lncRNA transcribed from AGAP2, is involved in various cancer types. However, the clinical significance, biological roles and regulatory mechanisms of AGAP2-AS1 in epithelial ovarian cancer (EOC) have not been thoroughly elucidated to date. In this study, we demonstrated the expression pattern and biological roles of AGAP2-AS1 in EOC. Clinically, AGAP2-AS1 expression was decreased in EOC tissues compared to that in the controls. Low expression of AGAP2-AS1 was associated with advanced FIGO stage, high histological grade, serous subtype and lymph node metastasis in patients with EOC. AGAP2-AS1 inhibited cell migration, invasion and proliferation *in vitro*. AGAP2-AS1 suppressed tumor growth* in vivo*. Mechanistically, AGAP2-AS1 inhibited cell metastasis and proliferation by downregulating KRAS, FGFR4, and CTSK and suppressing epithelial-mesenchymal transition. In conclusion, we provide the first evidence for the tumor-suppressing effect of AGAP2-AS1 in EOC and demonstrate that AGAP2-AS1 may represent a promising therapeutic target for EOC patients.

## Introduction

Ovarian cancer (OC) is the most lethal gynecological cancer and the fifth most common cause of cancer-related death among women 1. Epithelial ovarian cancer (EOC) accounts for the majority of OC and is characterized by widespread and rapid metastasis in the peritoneal cavity. Despite aggressive treatment, the prognosis of EOC patients remains poor due to tumor metastasis and recurrence. Therefore, there is a demand for new insights into EOC and for research characterizing the molecular mechanisms underlying EOC progression.

Recently, long noncoding RNAs have emerged as crucial functional molecules in various cancers 2-5. Among these lncRNAs, antisense noncoding RNAs, a class transcribed from the antisense strand of transcriptional units, are involved in the regulation of protein-coding genes through cis or trans- mechanisms. Most recently, AGAP2-AS1, an antisense long noncoding RNA that has been found to serve important functions in tumorigenesis and metastasis 6,7, has become the focus of our attention. AGAP2-AS1 is located on chromosome 12q14.1 and consists of 1567 nucleotides. This gene is on a locus with AGAP2 and may function as a co-regulator of AGAP2 8-10. It is now widely accepted that AGAP2 regulates cell migration 11, and overexpression of AGAP2 causes intracellular redistribution of activator protein 1 (AP- 1) 12, which is an important modulator in several carcinomas, such as breast 13,14 and endometrial carcinomas 15, ovarian cancer 16, colorectal cancer 17, gastric cancer 18, acute myeloid leukemia 19 and anaplastic large cell lymphoma 20. Despite these findings, however, the relationship between AGAP2-AS1 and AGAP2 has not been determined to date, and no published studies have investigated the clinical significance or biological function of AGAP2-AS1 in EOC.

In the present study, we examined the expression of AGAP2-AS1 in EOC tissues and ovarian cancer cell lines and analyzed the correlation between AGAP2-AS1 levels and clinicopathological features. Additionally, we investigated the functional impact of AGAP2-AS1 on tumor migration, invasion and proliferation during EOC progression through a series of *in vitro* and *in vivo* assays. Moreover, we also explored the molecular events that occurred downstream of AGAP2-AS1 involvement in EOC progression. These data provide novel insights into the role of AGAP2-AS1 in the progression of EOC and identify a potential therapeutic target in patients with EOC.

## Materials and Methods

### Tissue samples

Eighty EOC tissue samples were obtained from patients undergoing surgical treatment, and ten samples of normal ovarian surface epithelial (NOSE) tissue were collected as controls at the Obstetrics and Gynecology Hospital of Fudan University, China. Both written informed consent of patients and approval of the ethics committee were obtained for our study. A diagnosis of EOC was determined histologically by experienced pathologists. The 80 patients with EOC underwent standard ovarian cancer therapeutic approaches according to the guidelines, which included staging surgery and tumor cytoreductive surgery (debulking) surgery. Patients with borderline ovarian tumors or patients complicated with other gynecological tumors were excluded. No patients who had received preoperative treatment, such as radiotherapy, chemotherapy or hormonal therapy, were included in our studies. Ten NOSE tissue samples were obtained from participants diagnosed with uterine fibroids scheduled to undergo hysterectomy and oophorectomy. Patients with ovarian cysts or a history of ovarian surgery were excluded. The patients' clinicopathological data were collected by reviewing medical records.

### Cell lines and cell culture

Human ovarian cancer cell lines (SKOV3-ip, OVCAR3, HO8910, HEY, ES2) were purchased from the American Type Culture Collection (Manassas, VA, USA). All cell lines were maintained in RPMI-1640 medium (Gibco, MD, USA) containing 10% fetal bovine serum (FBS; Gibco, CA, USA) and 100 units/ml penicillin-streptomycin (Thermo Fisher Scientific, CA, USA) in a humidified 5% CO_2_ incubator at 37 °C. All cell lines were passaged for fewer than 6 months in our laboratory after resuscitation.

### Lentiviral vector production and establishment of stable AGAP2-AS1-knockdown or AGAP2-AS1-overexpression tumor cells

The recombinant AGAP2-AS1-shRNA-1/AGAP2-AS1-shRNA-2/AGAP2-AS1-overexpression lentivirus and the negative control lentivirus were prepared and titered to 10^9^ TU/ml (transfection unit). To obtain a stable AGAP2-AS1-knockdown cell line, SKOV3.ip/OVCAR3 cells were seeded in six-well dishes at a density of 3 × 10^3^ cells per well. The cells were then infected with virus at the same titer with 5μg/ml polybrene (GenePharma, shanghai, China) on the following day. Approximately 72 h after viral transfection, the culture medium was replaced with selection medium containing 2 μg/ml puromycin. The cells were then cultured for at least 14 days. The puromycin-resistant cell clones were isolated, amplified in medium containing 1 μg/ml puromycin for seven to nine days and transferred to a medium without puromycin. The clones were designated LV (AGAP2-AS1- knockdown) cells or NC (negative control) cells. The target sequences for AGAP2-AS1 shRNAs were 5′- GCTCTGTTCCCTCACGCTTAC (AGAP2-AS1-shRNA-1) and 5′- GGCATTCACTACACTCCCTAG -3′ (AGAP2-AS1-shRNA-2). The efficiency of AGAP2-AS1 knockdown was confirmed via quantitative real-time polymerase chain reaction (qRT-PCR).

### RNA isolation, reverse transcription, and qRT-PCR

Total RNA was extracted using TRIzol reagent (Invitrogen, CA, USA). RNA was reverse transcribed into cDNA using a Prime-ScriptTM one step RT-PCR kit (TAKARA, Dalian, China). AGAP2-AS1 expression levels were measured with qPCR in quadruplicate using SYBR Green PCR Master Mix (TAKARA, Dalian, China) on an ABI StepOne Real-Time PCR machine (Applied Biosystems, CA, USA). GAPDH was used as an endogenous standard. The sequences of the primers designed by Shanghai Engineering Biotech Corp (Shanghai, China) are listed as follows: AGAP2-AS1 forward primer, 5'- TACCTTGACCTTGCTGCTCTC-3' and reverse primer, 5'- TGTCCCTTAATGACCCCATCC-3'; and GAPDH forward primer, 5'- GGGAAGGTGAAGGTCGGAGT-3' and reverse primer, 5'- GGGGTCATTGATGGCAACA-3'.

### Cell migration and invasion assays

Migration and invasion of EOC cells were evaluated *in vitro* using a modified Boyden chamber Transwell system (Corning, MA, USA). Twenty-four hours after transfection, cells were seeded in Transwell plates for further experiments. For migration, the upper chamber was plated with 5× 10^4^ cells in 200 μL serum-free RPMI 1640, and the lower chamber was filled with 500 μL RPMI 1640 with 10% fetal bovine serum. After 24 h of incubation at 37°C, the cells on the upper surface of the filter were removed using cotton swabs. The migrated cells that remained on the bottom surface were fixed with methanol for 30 minutes and then stained with 0.1% crystal violet for 10 minutes. The number of cells in five random fields of each chamber was counted and imaged using a digital microscope (magnification, × 200). The average number of cells was calculated.

For the Matrigel invasion assay, the infected cells (1 × 10^5^) were plated in the top chamber of Transwell assay inserts (Corning, MA, USA) with a Matrigel-coated membrane containing 8 μm pores in 200 μL of serum-free RPMI 1640 medium. The assays were conducted in triplicate.

### Cell proliferation assays

The effect of AGAP2-AS1 on cell proliferation was determined using real-time cellular analysis (RTCA). Twenty-four hours after transfection, cells (5 × 10^3^ cells/well) were seeded into several E-Plate 16 (ACEA Biosciences Inc., USA) for proliferation assays according to the manufacturer's recommendations. The plates were kept in a cell incubator at 37 °C and 5% CO_2_ for 4-6 days. The cell index and growth curves were automatically recorded on an xCELLigence RTCA System (Roche, USA).

### Microarray analysis

Seventy-two hours after transfection of AGAP2-AS1 shRNA2 lentivirus or NC lentivirus, total RNA from SKOV3.ip and OVCAR3 cells was extracted using TRIzol reagent (Invitrogen, CA, USA). Then, the RT^2^ ProfilerTM PCR Array Human Tumor Metastasis Array (PAHS-028Z) was used to identify the differentially expressed genes of AGAP2-AS1 knockdown (KD) and control samples (NC). Differentially expressed genes, Gene Ontology (GO) and KEGG pathway analyses were used to investigate the functional significance of the molecular changes.

### Western blot assays

To analyze the expression of genes downstream of AGAP2-AS1, Western blot analyses were performed using the following primary antibodies: rabbit anti-human KRAS (Abcam; 1:200); anti-FGFR4 (Abcam; 1:1000); anti-CTSK (Abcam; 1:1000); anti-E-cadherin (Abcam; 1:50); anti-vimentin (Abcam; 1:2000) and anti-GAPDH (Abcam; 1:10000). Briefly, stimulated cells were lysed with RIPA buffer containing protease inhibitors (Roche, CA, USA); 20-30 μg samples of the lysates were separated on 10% SDS-PAGE gels and transferred to PVDF membranes. The membranes were incubated with primary antibodies overnight at 4 °C. The primary antibody incubation was followed by incubation with an HRP-conjugated secondary antibody. The bound antibodies were detected using an ECL kit (Tangon, shanghai, China).

### In vivo experiment

All animal studies were approved by the Institutional Animal Care and Use Committee of the Fudan University. Twelve female athymic Balb/c nude mice (aged 4 to 6 weeks, weighing 14-18 g) were purchased from Department of Laboratory Animal, Fudan University, housed in a pathogen-free animal facility and randomly assigned to the negative control lentivirus (SKOV3.ip-NC) group or the AGAP2-AS1- knockdown (SKOV3.ip-KD) group (six mice per group). A total of 10^7^ SKOV3.ip cells were subcutaneously injected into each mouse. The engrafted mice were inspected weekly for signs of distress or changes in weight and euthanized four weeks after the subcutaneous injection of the tumor cells.

### Statistical analysis

Student's t-test (two-tailed) was performed to analyze the *in vitro* data using GraphPad software. The relationship between AGAP2-AS1 expression levels and clinical parameters was assessed with the Pearson's correlation coefficients. All data are presented as the mean ± SD. *P*-values less than 0.05 were considered to be statistically significant.

## Results

### AGAP2-AS1 expression decreases in epithelial ovarian cancer tissues and correlates with EOC progression

**Figure 1:**
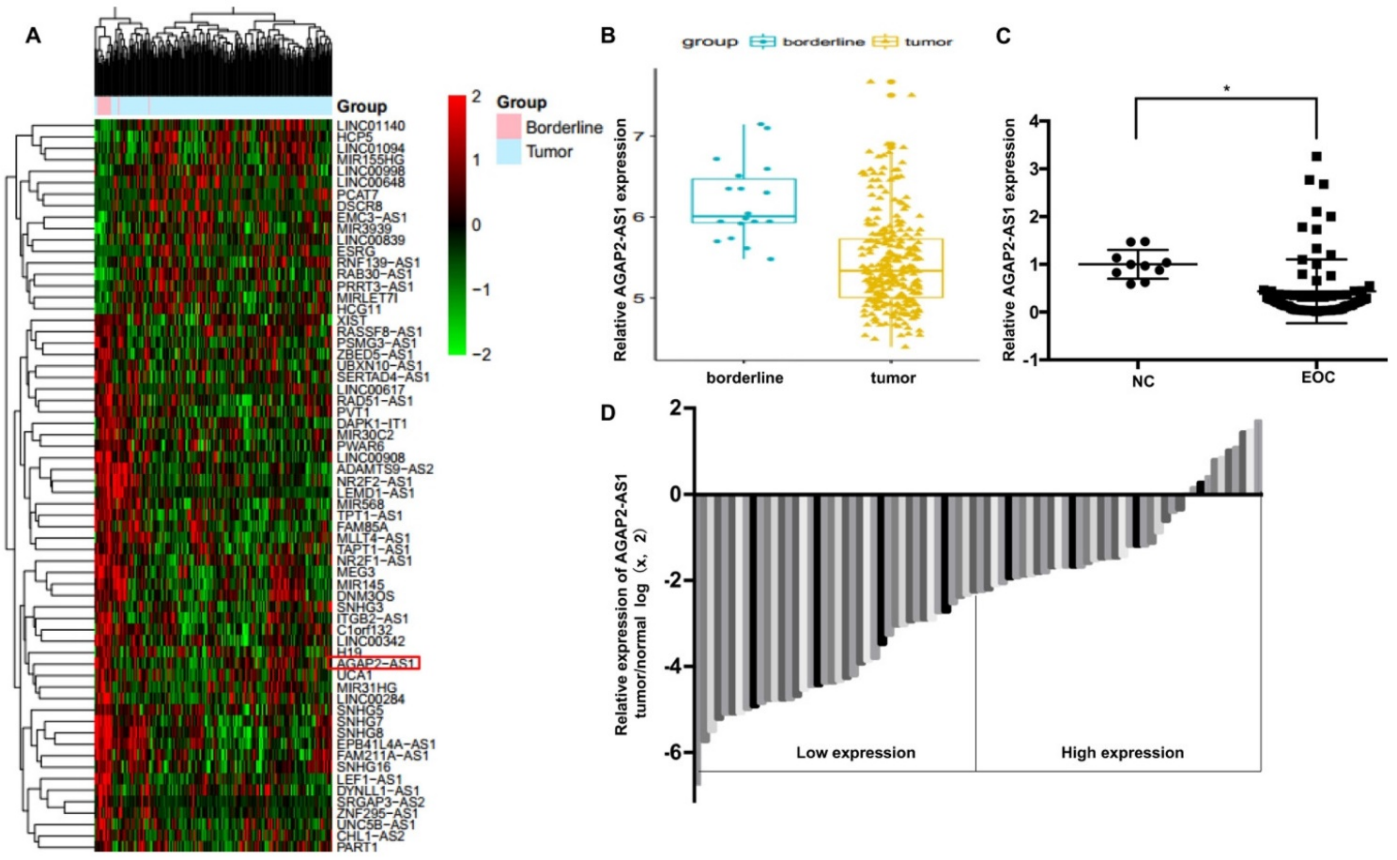
** AGAP2-AS1 was downregulated in EOC tissues.** (**A**) Differentially expressed lncRNAs between ovarian cancer (OC) tissues (n=267) and ovarian borderline tumors (n=18). (**B**) AGAP2-AS1 decreased in OC tissues compared to ovarian borderline tumors (*P* < 0.05, Student's t-test). (**C**) AGAP2-AS1 was downregulated in epithelial ovarian cancer samples (n=80) compared to that in normal ovarian epithelial tissues (n=10) (*P* < 0.05, Student's t-test). (**D**) EOC patients were divided into an AGAP2-AS1 high group (n = 40) and an AGAP2-AS1 low group (n = 40) using the median value of the AGAP2-AS1 expression levels.

To identify the role of AGAP2-AS1 in EOC, we first analyzed the GSE9891 microarray data obtained from the GEO database and found that AGAP2-AS1 was significantly downregulated in the 267 ovarian cancer tissues compared to the 18 ovarian borderline tumors (Fig. 1A-1B). Furthermore, we detected the expression of AGPA2-AS1 in 80 EOC tissues. As shown in Fig. 1C, AGAP2-AS1 expression decreased in most (86.25%, 69/80) EOC tissues compared to that in the normal ovarian samples, indicating that AGAP2-AS1 might play a part in EOC progression. To explore the correlation between AGAP2-AS1 levels and EOC malignant features, 80 EOC patients were divided into the AGAP2-AS1 high group (n = 40) and the AGAP2-AS1 low group (n = 40) using the median value (Fig. 1D). We found that low AGAP2-AS1 levels were associated with advanced FIGO stage, high histological grade, lymph node metastasis and serous subtype (Table 1), indicating that AGAP2-AS1 negatively correlates with a high degree of malignancy of EOC. Collectively, these results suggested that AGAP2-AS1 might act as a tumor suppressor in EOC.

### AGAP2-AS1 inhibits EOC cell migration, invasion and proliferation

**Figure 2:**
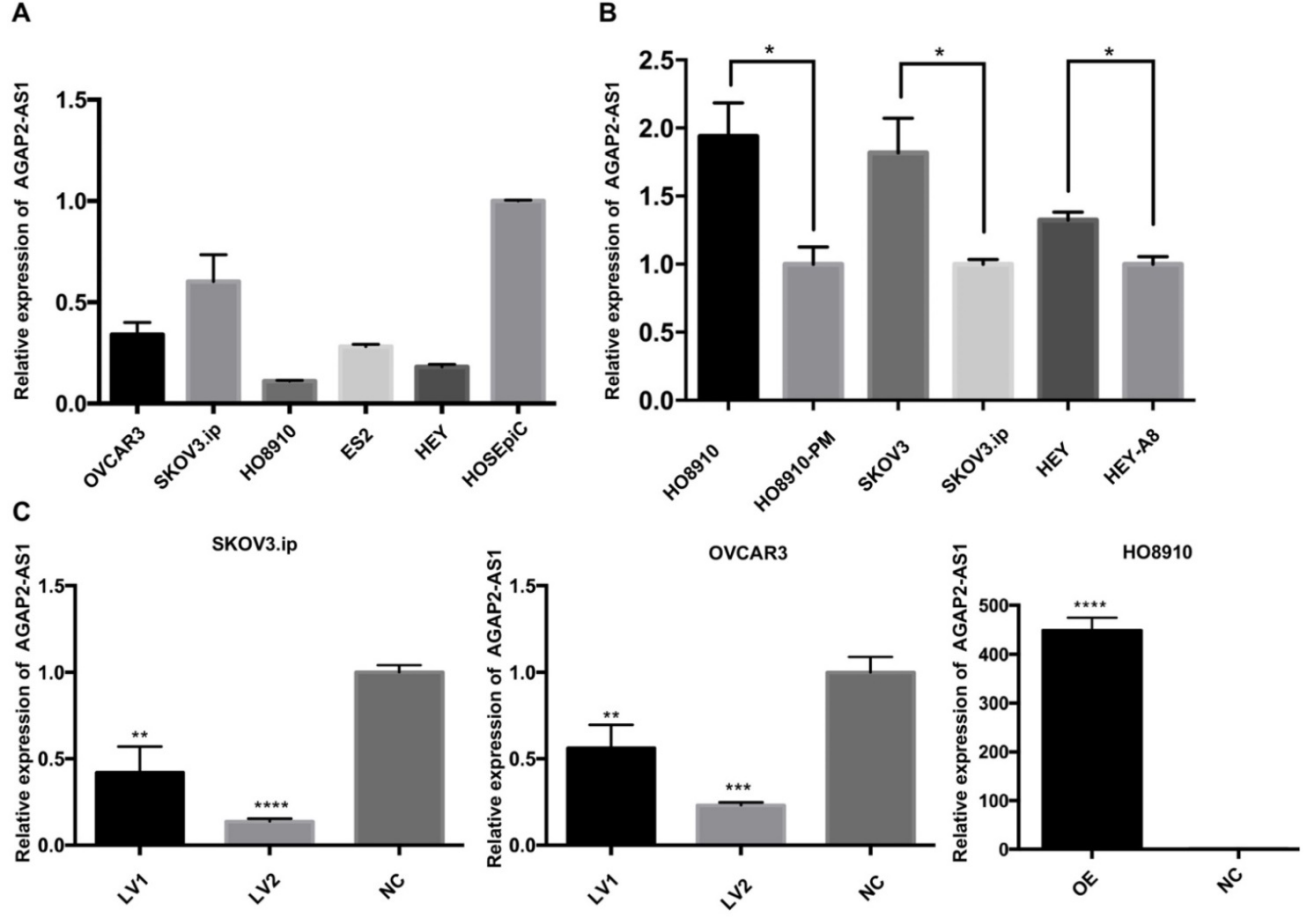
** Expression of AGAP2-AS1 in EOC cell lines.** (**A**) Relative AGAP2-AS1 expression in EOC cell lines. (**B**) AGAP2-AS1 was suppressed in highly invasive cell lines (HO8910-PM, SKOV3.ip, HEY-A8) compared to that in the controls (HO8910, SKOV3, HEY). (**C**) AGAP2-AS1 was significantly silenced/overexpressed by specific lentiviruses in SKOV3.ip, OVCAR3 and HO8910 cells and verified by qRT-PCR verification.

Based on the clinical observations, we hypothesized that downregulation of APAP2-AS1 promoted EOC progression. To this end, we measured the expression patterns of AGAP2-AS1 in EOC cell lines. The qRT-PCR results showed that AGAP2-AS1 was downregulated in the EOC cell lines (SKOV3.ip, OVCAR3, HO8910, HEY, ES2) compared with the normal human ovarian epithelial cell line (HOSEpiC) (Fig. 2A). Furthermore, the expression levels of AGAP2-AS1 in three pairs of cell lines (HO8910 vs HO8910-PM, SKOV3 vs SKOV3.ip, HEY vs HEY-A8) were detected, and AGAP2-AS1 expression was found to be decreased in highly invasive EOC cell lines (HO8910-PM, SKOV3.ip, HEY-A8) compared to their corresponding controls (HO8910, SKOV3, HEY) (Fig. 2B). These findings collectively suggested that AGAP2-AS1 might be involved in the aggressive biological behaviors of EOC cells.

**Figure 3:**
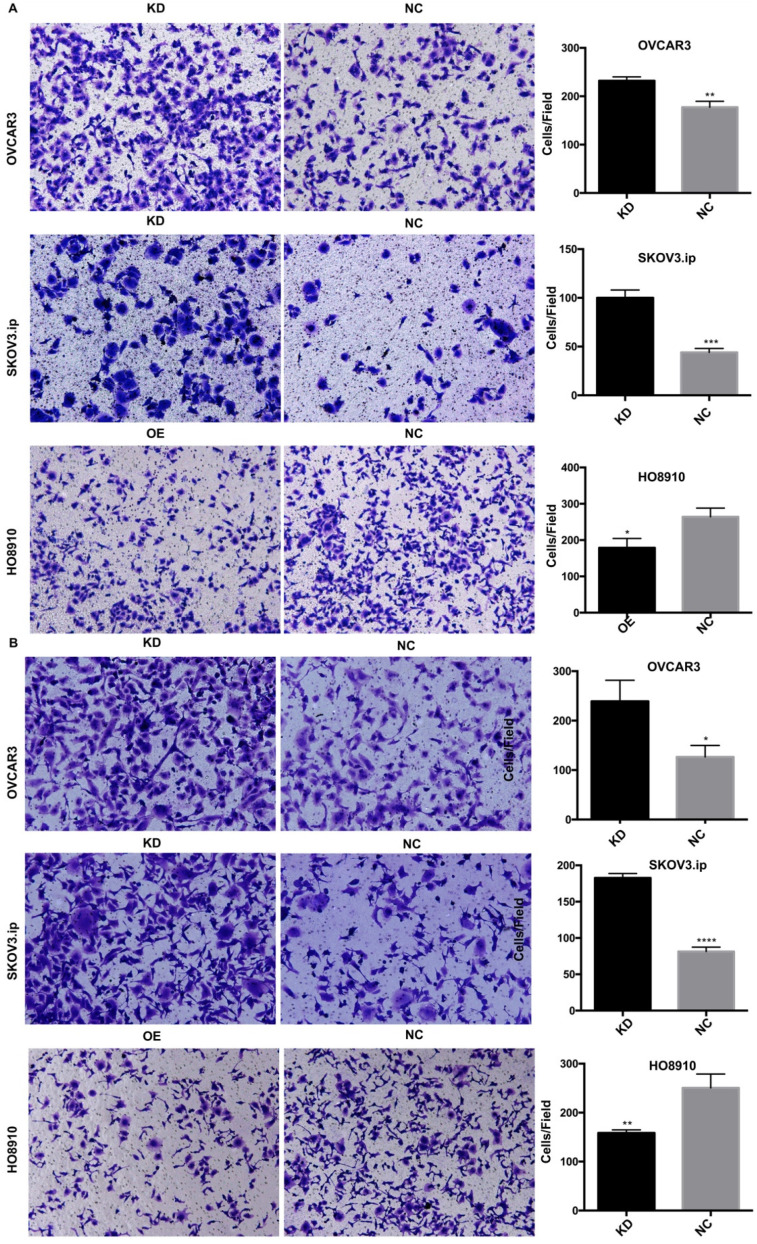
** AGAP2-AS1 regulated cell migration, invasion in SKOV3.ip, OVCAR3 and HO8910 cells.** (**A**) AGAP2-AS1 silencing promoted cell migration of SKOV3.ip and OVCAR3 cells, and AGAP2-AS1 overexpression inhibited cell migration of HO8910 cells. (**B**) A Matrigel invasion assay showed that knockdown of AGAP2-AS1 increased the invasive abilities of SKOV3.ip and OVCAR3 cells and that upregulation of AGAP2-AS1 inhibited the invasion of HO8910 cells.

**Figure 4:**
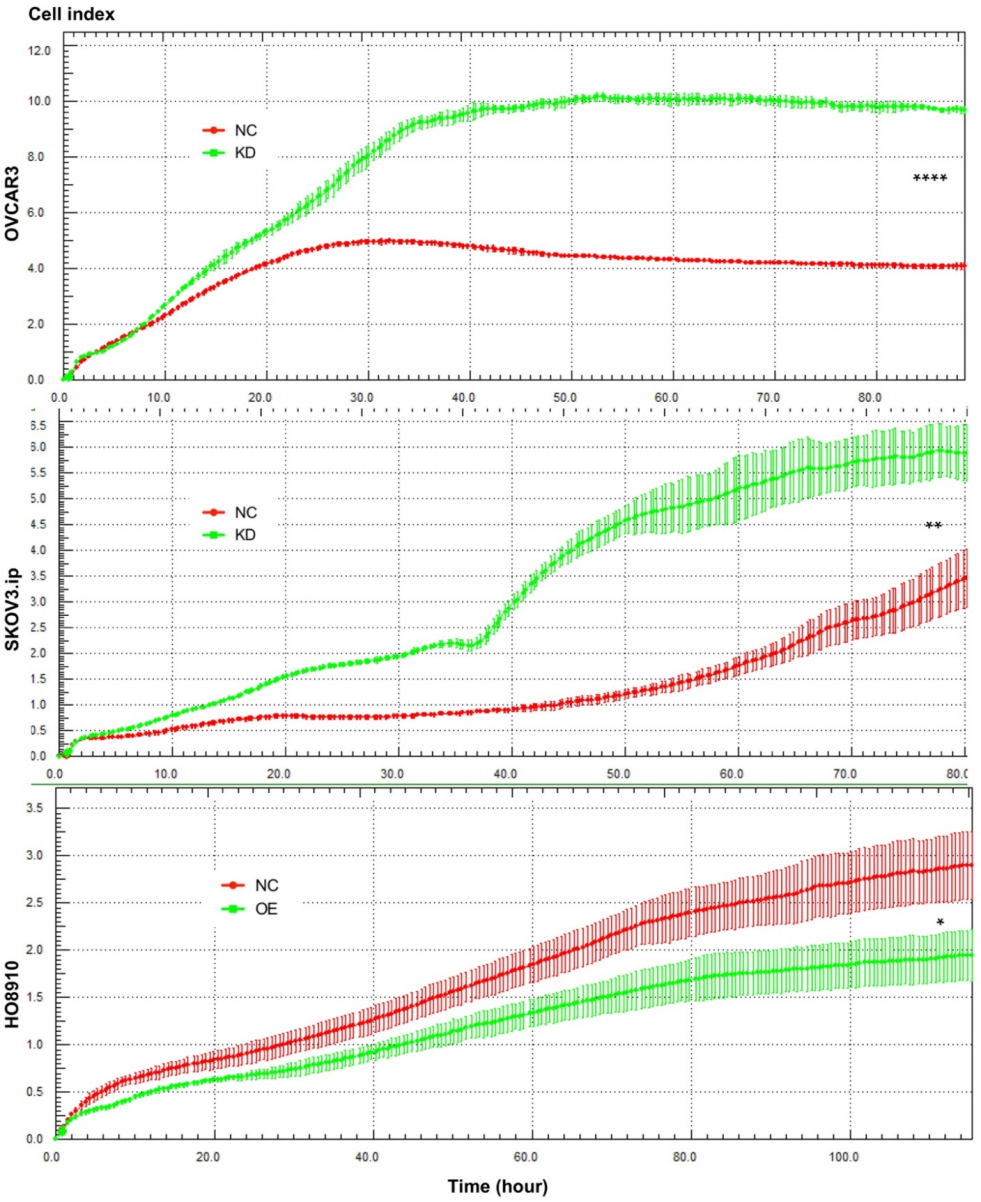
** AGAP2-AS1 regulated cell proliferation in SKOV3.ip, OVCAR3 and HO8910 cells.** Proliferation assays showed that downregulation of AGAP2-AS1 contributed to cell proliferation in SKOV3.ip and OVCAR3 cells, while upregulation of AGAP2-AS1 suppressed HO8910 cell growth. Each experiment was repeated in triplicate (n = 3). (The results are shown as the mean ± SD; *P < 0.05; **P < 0.01; ***P < 0.001; **** P < 0.0001; Student's t-test).

To investigate whether AGAP2-AS1 plays a role in cell migration, invasion and proliferation in EOC cells, AGAP2-AS1 was knocked down or overexpressed by lentivirus transfection. The results showed that the lentiviruses exerted a satisfactory silencing/overexpressing effect (Fig. 2C). LV2 showed a superior knockdown efficiency and was used for further tests. The migration assay showed that downregulation of AGAP2-AS1 promoted the migration of SKOV3.ip and OVCAR3 cells, while upregulation of AGPA2-AS1 suppressed HO8910 migration (Fig. 3A). The Matrigel invasion assay indicated that the suppression of AGAP2-AS1 expression significantly increased the invasive ability of SKOV3.ip and OVCAR3 cells. Conversely, overexpression of AGAP2-AS1 decreased the invasiveness of HO8910 cells (Fig. 3B). We therefore concluded that silencing AGAP2-AS1 promotes EOC cell migration and invasion *in vitro*. Additionally, we performed RTCA to determine whether AGAP2-AS1 is involved in cell proliferation. As expected, cell proliferation assays showed that AGAP2-AS1 silencing significantly promoted the proliferation of SKOV3.ip and OVCAR3 cells (Fig. 4). However, overexpressed AGAP2-AS1 has a slight suppressive effect on HO8910 cell growth. In summary, these results suggest that AGAP2-AS1 inhibits cell migration, invasion and proliferation *in vitro*.

### AGAP2-AS1 suppresses EOC growth in vivo

**Figure 5:**
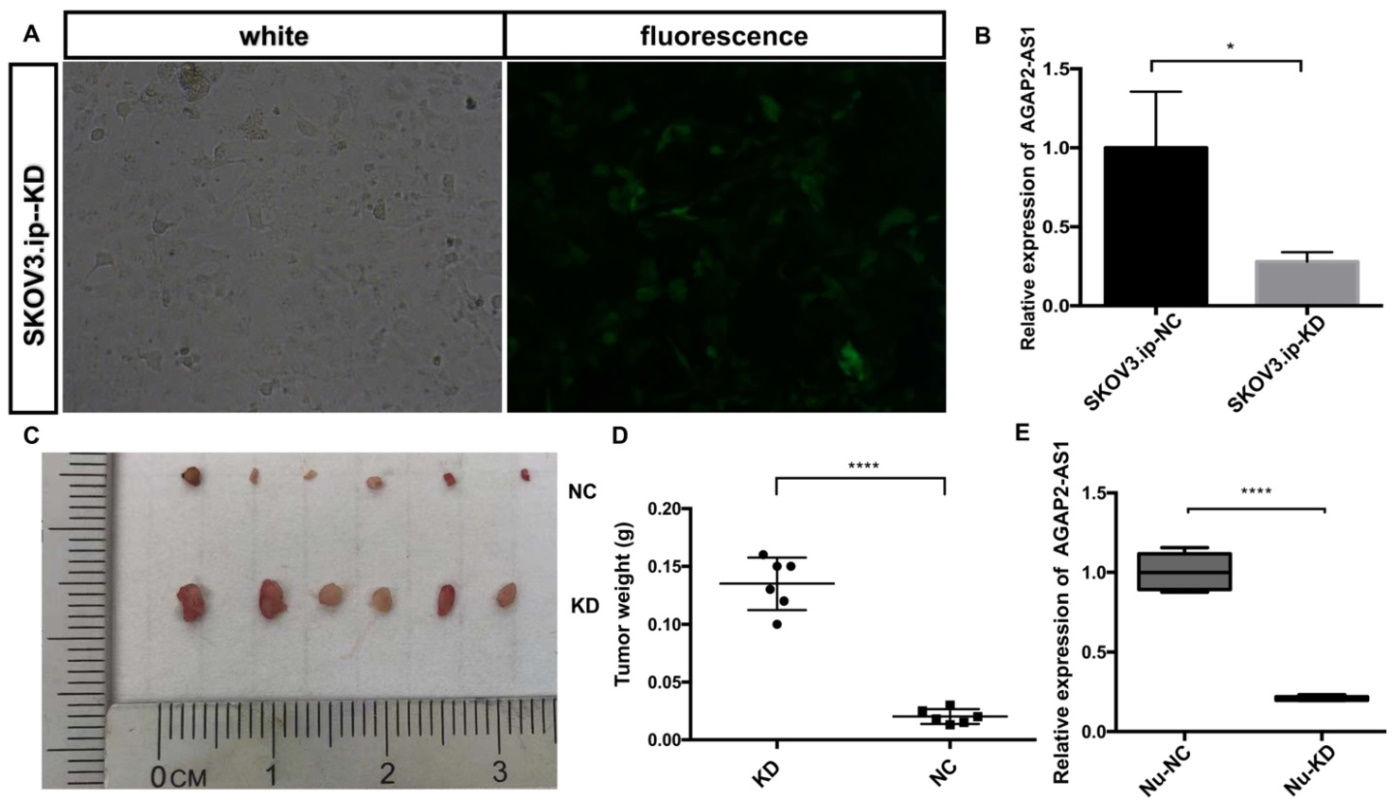
** AGAP2-AS1 knockdown promoted tumor growth *in vivo*.** (**A**) The efficiency of lentivirus transfection was visually assessed by microscopy. (**B**) The knockdown efficiency of SKOV3.ip-KD was normalized to SKOV3.ip-NC with qRT-PCR confirmation. (**C**) Tumor volumes in the LV-KD group were significantly larger than those in the LV-NC group. (**D**) Tumor weight in the LV-KD group was significantly increased compared with that in the LV-NC group. (**E**) The relative expression of AGAP2-AS1 in the Nu-KD group was significantly decreased compared with that in the Nu-NC group. (LV-KD: AGAP2-AS1-knockdown lentivirus cells, LV-NC: AGAP2-AS1-negative control lentivirus cells *P < 0.05; ****P < 0.0001 by Student's t-test).

To assess the effect of AGAP2-AS1 *in vivo*, a nude mouse xenograft model was established. SKOV3.ip cells with (KD) or without (NC) AGAP2-AS1 knockdown were injected subcutaneously into mice. The results showed that AGAP2-AS1 was effectively downregulated in SKOV3.ip-KD cells compared with that in SKOV3.ip-NC cells (Fig. 5A and B), and the silencing efficiency was confirmed after tumor removal (Fig. 5E). The downregulation of AGAP2-AS1 increased the volume of tumors (Fig. 5C). The tumor weight in the KD group was significantly elevated compared with the NC group (Fig. 5D). To summarize, these results indicate that silencing AGAP2-AS1 promoted tumor growth *in vivo*.

### AGAP2-AS1 suppresses EMT and dysregulates KRAS, CTSK and FGFR4 in EOC progression

**Figure 6:**
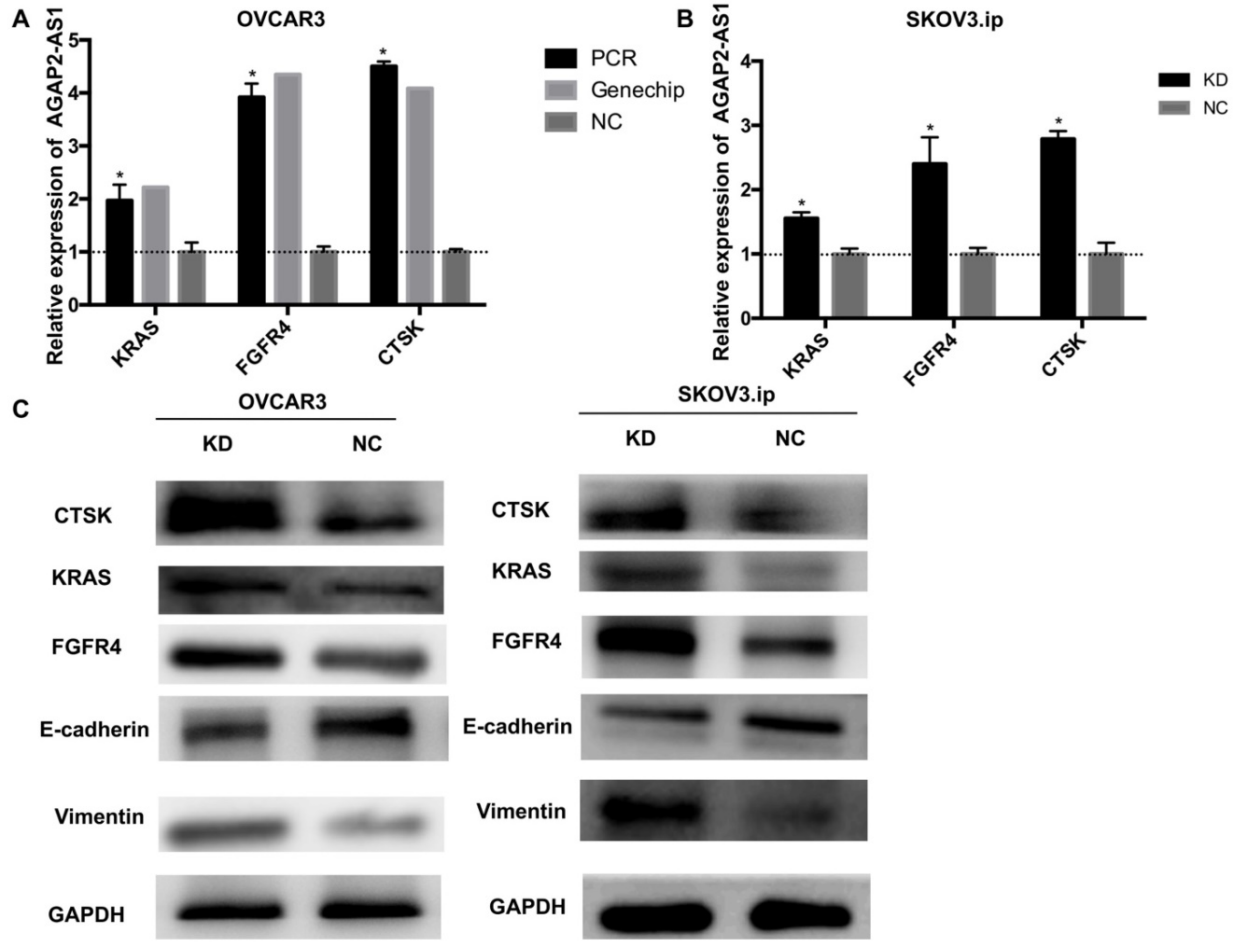
** AGAP2-AS1 knockdown regulated KRAS, CTSK, and FGFR4 expression in SKOV3.ip and OVCAR3 cells and induced epithelial-mesenchymal transition.** (**A**) KRAS, FGFR4, and CTSK were significantly upregulated in SKOV3.ip and OVCAR3 cells after AGAP2-AS1 silencing, which was consistent with the PCR array results. (**B**) The protein levels of KRAS, FGFR4, and CTSK were detected by Western blotting after AGAP2-AS1 knockdown. After AGAP2-AS1 knockdown, E-cadherin levels were reduced, and vimentin levels were increased. All results are shown as the mean ± SD and are representative of three independent experiments. (*P < 0.05, Student's t-test).

To further illuminate the possible mechanisms through which AGAP2-AS1 contributed to EOC progression, gene expression profiles of OVCAR3-KD cells were compared with those of OVCAR3-NC cells using real-time PCR array analysis (PAHS-028Z). The data were further validated by qRT-PCR, and the results showed that KRAS, CTSK, and FGFR4 were markedly dysregulated (>2-fold) after AGAP2-AS1 silencing in both OVCAR3 and SKOV3.ip cells (Fig. 6A-B). Additionally, consistent with the array results, increased KRAS, CTSK, and FGFR4 protein levels were detected in both SKOV3.ip and OVCAR3 cells after AGAP2-AS1 knockdown (Fig. 6C). Furthermore, E-cadherin was downregulated and vimentin was up-regulated after AGAP2-AS1 knockdown (Fig. 6C), suggesting that AGAP2-AS1 suppressed epithelial-mesenchymal transition. Taken together, these data indicate that AGAP2-AS1 regulates EOC cell migration and invasion by suppressing EMT and decreasing cell proliferation by regulating KRAS, CTSK and FGFR4.

## Discussion

LncRNA, as a major component of noncoding RNAs, has attracted considerable attention in recent years. AGAP2-AS1 is an antisense RNA that co-regulates the function of AGAP2 8-10. It is now widely accepted that AGAP2 regulates focal adhesion kinase (FAK) activity and focal adhesion disassembly during cell migration 11. In addition, overexpression of AGAP2 induced AP1 and was associated with metastasis and proliferation in various tumors 20. Recently, AGAP2-AS1 was reported to be involved in tumorigenesis and metastasis 6,7. However, no studies have reported the presence of AGAP2-AS1 in ovarian cancer, and no published studies have investigated the clinical significance or biological function of AGAP2-AS1 in EOC.

In our study, we found that AGAP2-AS1 expression significantly decreased in EOC tissues compared with that in noncancerous tissues. With a thorough analysis of clinical data, we found that low expression levels of AGAP2-AS1 are associated with advanced FIGO stage, high histological grade, lymph node metastasis and serous histological subtype. These findings indicate that lower expression of AGAP2-AS1 may promote aggressiveness and metastasis of EOC and may serve as a tumor suppressor in patients with EOC.

Previous studies showed that upregulation of AGAP2-AS1 promoted cell proliferation and metastasis in pancreatic cancer, glioma, gastric cancer and non-small-cell lung cancer 6,7,21,22. Overexpression of AGAP2-AS1 could cause trastuzumab resistance in breast cancer 23. However, in our study, the results showed that AGAP2-AS1 is downregulated in highly invasive EOC cell lines compared to the controls. Knockdown of AGAP2-AS1 promoted the migration, invasion and proliferation of SKOV3.ip and OVCAR3. We found that mice injected with AGAP2-AS1-KD cells developed significantly larger tumor implants compared to the controls. Silencing of AGAP2-AS1 led to a dramatic decrease in tumor weight. These results collectively demonstrated that AGAP2-AS1 is a potential tumor suppressor in EOC and that knockdown of AGAP2-AS1 contributes to EOC proliferation and metastasis. Our results are in contrast to the previously reported roles of AGAP2-AS2 in other cancers and indicate the complicated roles of lncRNAs in cancers.

As is well-known, long antisense noncoding RNAs were found to serve as regulators of their sense counterparts. For example, antisense noncoding RNA p15AS induced p15 silencing and promoted cell growth in leukemia 24. In breast cancer, RAD51-AS1 exhibited an opposite effect in regulating the expression of RAD51 and induced RAD51-dependent double-strand break repair in malignancy 25. Consistent with these findings, we found that the expression of AGAP2-AS1 in EOC tissues is negatively correlated with that of AGAP2. AGAP2 is known to be a proto-oncogene and is involved in the activity of AKT, NF-κB, p53, AMPK, and FAK 11,26-29. Furthermore, researchers found that AGAP2 promoted cell proliferation, migration, and invasion and suppressed cell apoptosis 30,31. As the antisense lncRNA of AGAP2, AGAP2-AS1 was found to conversely inhibited cell proliferation and metastasis in EOC cell lines, and low expression of AGAP2-AS1 was correlated with tumor malignant features, indicating that AGAP2-AS1 is a potential tumor suppressor of EOC.

The contradictory roles of lncRNAs in different cancers open up new possibilities for achieving a deep understanding of their functions and research methods. For example, MALAT1, which has previously been described as a metastasis-promoting lncRNA, has recently been found to suppress breast cancer metastasis 32. The opposite functions of lncRNA in different cancers may be attributed to the tissue-specific expression pattern and regulatory diversities. In addition, different knockdown techniques may lead to different results.

To date, our understanding of the molecular mechanisms of lncRNAs in tumor progression is far from enough. Previous studies have demonstrated that lncRNAs promote cancer metastasis and proliferation by regulating protein-coding genes. As expected, our results demonstrated that the expression of EMT-related genes (FGFR4, CTSK, E-cadherin, Vimentin) and oncogenic genes (KRAS) were dysregulated by AGAP2-AS1 knockdown in EOC cell lines (SKOV3.ip and OVCAR3), implying that these genes participate in AGAP2-AS1-induced EOC progression.

However, further research is needed to elucidate the regulatory mechanisms underlying the relationship between AGAP2-AS1 and its target genes. In addition, studies with larger samples are required to explore the prognostic value of AGAP2-AS1 in EOC patients.

In conclusion, our data provide evidence for the involvement of AGAP2-AS1 in EOC proliferation and metastasis and suggest that AGAP2-AS1 may be a tumor suppressor in EOC. AGAP2-AS1-based therapeutic strategies aimed at upregulating suppressive lncRNAs may represent a promising alternative therapeutic approach for the treatment of patients with EOC.

## Figures and Tables

**Table 1:** Association of AGAP2-AS1 expression with clinicopathological variables in EOC patients

Variable	Low AGAP2-AS1	High AGAP2-AS1	*P*
	Expression, n (%)	Expression, n (%)	
	n=40	n=40	
**Age (years)**			
<50	13	12	0.809
≥50	27	28	
**Histological subtype**			
Serous	39	28	0.001
Other	1	12	
**FIGO stage**			
I-II	7	15	0.045
III-IV	33	25	
**Histological grade**			
High	39	31	0.007
Low	1	9	
**Residual tumor diameter (cm)**			0.396
<1	36	38	
≥1	4	2	
**Lymph node metastasis**			
Absent	20	29	0.039
Present	20	11	
**CA125 level (U/ml)**			
<600	18	20	0.654
≥600	22	20	
**Ascites**			
<100	21	24	0.499
≥100	19	16	

NOTE: The median value of AGAP2-AS1 expression was used as a cut-off for the low/high AGAP2-AS1 expression groups.

## Abbreviations

AP-1: activator protein 1
CTSK: cathepsin K
EMT: epithelial-mesenchymal transition
EOC: epithelial ovarian cancer
FGFR4: fibroblast growth factor receptor 4
FBS: fetal bovine serum
FIGO: International Federation of Gynecology and Obstetrics
GO: Gene Ontology
KEGG: Kyoto Encyclopedia of Genes and Genomes
lncRNA: long noncoding RNA
NOSE: normal ovarian surface epithelial tissues
OC: ovarian cancer
RTCA: real-time cellular analysis
